# Opening up our windows to the world

**DOI:** 10.1007/s40037-012-0004-5

**Published:** 2012-03-10

**Authors:** Olle ten Cate

**Affiliations:** University Medical Center, Utrecht, The Netherlands

The Board of the Netherlands Association for Medical Education (NVMO) is extremely proud to welcome the readers of this first issue of Perspectives on Medical Education to take part in one of the Association’s most important channels of communication.

Since its inception 40 years ago, in 1972, NVMO has avidly promoted the two major causes and targets of its existence and mission: development and research of medical education. After about 10 years, an independent Netherlands Journal of Medical Education (TMO) was launched, and after about 20 years the first national conference was held, two major platforms for communication with and for the medical education community at large. As is reflected in its membership of 1,200 and rising, the Association has flourished nationally and in recent years has embarked on international activities. Since 2008, many NVMO Special Interest Groups have contributed to an NVMO guest series in our esteemed sister-journal Medical Teacher, and numerous NVMO members have published in international journals in the past decades. We are now ready for the next step.

On this occasion we wish to compliment the Editor and Editorial Board of our Journal on their vision, planning and execution of the transition of TMO to PME. We have already witnessed enthusiasm in the past year in a small international group of expert colleagues who were informed about this initiative, and we trust that PME will meet with similar enthusiasm among international readers and authors in the years to come. NVMO is more than happy to share and exchange ideas and research in the broad field of medical education. Not only does NVMO include all health professions education in its mission, ‘Netherlands’ has long included our Dutch speaking Flemish neighbours. We are now ready to officially welcome the international community.

